# Violet Laser Diode Enables Lighting Communication

**DOI:** 10.1038/s41598-017-11186-0

**Published:** 2017-09-05

**Authors:** Yu-Chieh Chi, Yu-Fang Huang, Tsai-Chen Wu, Cheng-Ting Tsai, Li-Yin Chen, Hao-Chung Kuo, Gong-Ru Lin

**Affiliations:** 1 Graduate Institute of Photonics and Optoelectronics, National Taiwan University (NTU), No. 1, Sec. 4, Roosevelt Road, Taipei, 10617 Taiwan, R.O.C.; 2 Department of Photonics, National Sun Yat-Sen University (NSYSU), No. 70, Lienhai Rd., Kaohsiung, 80424 Taiwan, R.O.C.; 3 Department of Photonics, National Chiao Tung University (NCTU), No. 1001, Ta Hsueh Rd, Hsinchu, 30050 Taiwan, R.O.C.

## Abstract

Violet laser diode (VLD) based white-light source with high color rendering index (CRI) for lighting communication is implemented by covering with Y_3_Al_5_O_12_:Ce^3+^ (YAG:Ce) or Lu_3_Al_5_O_12_:Ce^3+^/CaAlSiN_3_:Eu^2+^ (LuAG:Ce/CASN:Eu) phosphorous diffuser plates. After passing the beam of VLD biased at 70 mA (~2*I*_*th*_) through the YAG:Ce phosphorous diffuser, a daylight with a correlated color temperature (CCT) of 5068 K and a CRI of 65 is acquired to provide a forward error correction (FEC) certified data rate of 4.4 Gbit/s. By using the VLD biased at 122 mA (~3.5*I*_*th*_) to excite the LuAG:Ce/CASN:Eu phosphorous diffuser with 0.85-mm thickness, a warm white-light source with a CCT of 2700 K and a CRI of 87.9 is obtained at a cost of decreasing transmission capacity to 2.4 Gbit/s. Thinning the phosphor thickness to 0.75 mm effectively reduces the required bias current by 32 mA to achieve the same CCT for the delivered white light, which offers an enlarged CRI of 89.1 and an increased data rate of 4.4 Gbit/s. Further enlarging the bias current to 105 mA remains the white-light transmission capacity at 4.4 Gbit/s but reveals an increased CCT of 3023 K and an upgraded CRI of 91.5.

## Introduction

With the rapid development of self-sustainable green-smart houses, visible light communication (VLC), optical wireless communication (OWC) and light fidelity (Li-Fi) have attracted much attention as indoor users always demand huge data capacity and multi-functional lighting control during daily life^1, 2^. In contrast to traditional Wi-Fi and fiber-optic communications, the VLC system is a complementary system with advantages of license free, electromagnetic immunity and safe communication in free space. The VLC has found its suitable application scenarios in mobile connection, indoor positioning, vehicle transportation, targeted communication, underwater resource exploration and hospital/healthcare applications^3–13^. At this stage, some of the proof-of-concept VLC prototypes have already been implemented for commercial applications. By using light-emitting diodes (LEDs) to communicate with the camera of smartphones, several worldwide companies including Bytelight, Target, Emart and Royal Philips NV have successfully guided shoppers to goods based on their position in retail stores^14–17^. Besides, the Disney Research has particularly focused on enabling the visible light for the interaction between toys, magic wands and princess dresses, and toys and smartphones^18–20^. Even in Taiwan, the VLC based intelligent medical care system has been developed in hospitals through the cooperation with the Industrial Technology Research Institute (ITRI), which quickly and precisely realizes the positioning sensing of medical personnel and the wireless streaming of therapeutic data. Recently, the motor companies also signed an agreement to cooperate with universities for the vehicular VLC development project with improving driving safety and upgrading road-utilization rate^21^. According to the Grand View Research’s report, the expected revenue of the global VLC market will reach up to USD 101.30 billion by 2024^22^.

To achieve data rate up to Gbit/s for white-light LED based VLC systems, Khalid *et al*.^23^ and Wu *et al*.^24^ individually employed the rate-adaptive discrete multitone (DMT) and the carrierless amplitude phase (CAP) modulation schemes, respectively. Cossu *et al*.^25^ and Wu *et al*.^26^ further realized the wavelength division multiplexing (WDM) based VLC systems to upgrade the data rates to 3.4 and 3.22 Gbit/s, respectively. Later on, Chun *et al*. demonstrated a phosphorescent micro-LED (μLED) based VLC at 1.68 Gbit/s^27^. In particular, Wang *et al*. implemented the bidirectional VLC with four visible LEDs for 5.6-Gbit/s downlink and one infrared LED for 1.5-Gbit/s uplink over 1.5 m in free space^28^. Manousiadis *et al*. encapsulated the GaN μLEDs with polymer based red/green/blue (RGB) color converters for VLC with aggregated data rate of 2.3 Gbit/s^29^. By using the CAP modulation with a recursive least square (RLS) based adaptive equalizer, a RGB-LED based WDM-VLC with aggregated data rate of 4.5 Gbit/s over 1.5-m free-space transmission was demonstrated by Wang *et al*.^30^. The high-order CAP modulation and hybrid post equalization additionally helps the red/blue/green/yellow (RBGY)-LED based WDM-VLC to approach 8-Gbit/s free-space transmission over 1 m^31^. More recently, Bamiedakis *et al*. employed a μLED with 3-tap feed-forward pre-equalized 4-level pulse amplitude modulation (PAM-4) to demonstrate a 2-Gbit/s VLC link over 0.6 m^32^. Sun *et al*. fabricated an aluminum-doped zinc oxide (AZO) LED with maximal output power of 42 mW to carry 3-Gbit/s 32-QAM OFDM data at chip level^33^. By employing bit-rate adaptive OFDM scheme, Chun *et al*. used RC R-LED and B/G-μLEDs to demonstrate WDM-VLC transmission up to 11.28 Gbit/s^34^. The PAM-8 based phase-shifted (PS) Manchester coding proposed by Chi *et al*. enables the RGB-LED based WDM-VLC at 3.375 Gbit/s over 1 m^35^. Ferreira *et al*. used a B-μLED with *f*_*3dB*_ > 800 MHz for on-off-keying (OOK), PAM-4 and bit-loaded OFDM at 1.7, 3.4, and 5 Gbit/s, respectively^36^. Hsu *et al*. built up a 3 × 3 imaging multiple-input multiple-output (MIMO) VLC, in which the phosphor coated white-light LEDs can deliver 1-MHz bit-loaded OFDM data to achieve 1-Gbit/s transmission over 1 m^37^. With defining the LED based WDM-VLC channel spacing as 33 nm, Cui *et al*. performed 10-channel OOK data streaming up to 5.1 Gbit/s^38^ under indoor illumination standard. Lu *et al*. established a 2 × 2 polarization-multiplexing MIMO VLC system with RGB LEDs to achieve 6.36 Gbit/s over 1 m^39^. Shen *et al*. employed a 405-nm superluminescent diode (SLD) with 3-dB bandwidth of 807 MHz for 1.3-Gbit/s OOK transmission^40^. Islim *et al*. used a 400-nm violet μLED with *f*_*3dB*_ of only 655 MHz to carry the bit- and energy-loaded OFDM for VLC at 11.95 Gbit/s^41^.

As compared to the currently available LEDs with limited modulation bandwidth of several tens MHz^42–44^, the laser diodes (LDs) with higher coherence, narrower spectral linewidth and higher relaxation oscillation frequency is capable of handling data bandwidth up to several GHz^45, 46^, which is more suitable for next-generation lighting communications. Recently, Watson *et al*. used a BLD with 1.4-GHz modulation bandwidth for error-free OOK transmission at 2.5 Gbit/s^47^. Later on, a RLD based bidirectional 2.5-Gbit/s VLC over 20-km single-mode fiber (SMF) and 15-m free space was reported by Chen *et al*.^48^. With low-noise amplification and equalization at receiving end, Chang *et al*. demonstrated a 100-Gbit/s (12.5 Gbit/s/channel × 8 channels) MIMO-VLC over 5 m by employing vertical-cavity surface-emitting lasers (VCSELs)^49^. Tsonev *et al*. demonstrated a 4-Gbit/s OFDM based VLC over 2.88 m with RGB LDs^50^. By using a BLD combined with a remote phosphor, Chun *et al*. demonstrated a white-light VLC to support 6.52-Gbit/s data rate^51^. Janjua *et al*. demonstrated a RGB LDs based 20-cm-long WDM-VLC with data rate beyond 4 Gbit/s^52^, and the proposed white light exhibits a correlated color temperature (CCT) of 5835 K. Lee *et al*. demonstrated a VLC by using a high-power 450-nm GaN LD with 2.6-GHz bandwidth to achieve OOK transmission at 4 Gbit/s^53^. By employing a TO-38-can packaged GaN BLD with *f*_*3dB*_ ≤0.9 GHz, Chi *et al*. proposed a free-space VLC with 64-QAM OFDM transmission at 9 Gbit/s over 5 m in free space^54^. For both indoor white-lighting and OWC, Durán Retamal *et al*.^55^ and Chi *et al*.^56^ demonstrated phosphorous diffuser covered BLD based white-lighting VLCs to support 4- and 5.2-Gbit/s transmissions, respectively. Shen *et al*. presented an integrated waveguide LD/modulator module enabling 1-Gbit/s OOK data modulation at 448 nm^57^. Lu *et al*. encoded bit- and power-loaded OFDM onto a 682-nm VCSEL with *f*_*3dB*_ of only 1 GHz for 11.1-Gbit/s VLC over 1.2 m^58^. With the same VCSEL, Yeh *et al*. further demonstrated a point to multi-point (P2M) VLC which simultaneously supports four end-users with 5.04-Gbit/s data rate over 2 m^59^. By mixing RGB laser beams, Wu *et al*. implemented a white-light source with a CCT of 8382 K and a color rendering index (CRI) of 54.4 to provide aggregated data rate up to 8.8 Gbit/s over 0.5 m in free space^60^.

Note that either the RGB mixed lights^61, 62^ or the yellow phosphor combined blue light^63, 64^ has been widely realized for white-light generation. Therein the white light generated by illuminating the blue light through the yellow phosphor becomes a more compact and cost-effective solution than the RGB mixed white-light source with higher transmission capacity. Fortunately, such a drawback can be alleviated by carrying high-spectral-efficiency data formats such as high-level quadrature amplitude modulation (QAM)-OFDM^65, 66^. However, the BLD based white-light source inevitably suffers from insufficient CRI^67^ with the lack of violet color, thus limiting its applications in some high-end lighting circumstances such as museums, galleries and medical field. The use of violet LD (VLD) for exciting the fluorescent phosphor would be helpful, which enables the generation of white light with larger luminous efficiency and CRI than that demonstrated by using the BLD^68^. Nonetheless, the VLD based lighting communication has yet to be discussed to date.

In this work, for the first time, the phosphorous diffuser covered VLD at 405 nm is demonstrated for both white-lighting and QAM-OFDM data transmission in free space, in which two different fluorescent phosphors of Y_3_Al_5_O_12_:Ce^3+^ (YAG:Ce) and Lu_3_Al_5_O_12_:Ce^3+^/CaAlSiN_3_:Eu^2+^ (LuAG:Ce/CASN:Eu) are employed for comparison. At beginning, the VLD based point-to-point (PtP) VLC system for free-space transmission over 7 m is demonstrated. By exciting the phosphorous diffusers with the light of the VLD at different bias currents, the white-lighting performances including CCT, CRI and luminous flux are discussed. Furthermore, the optimization on allowable encoding bandwidth and transmission performance of the white light carried QAM-OFDM data over 0.5 m in free-space is performed.

## Results

**Figure 1:**
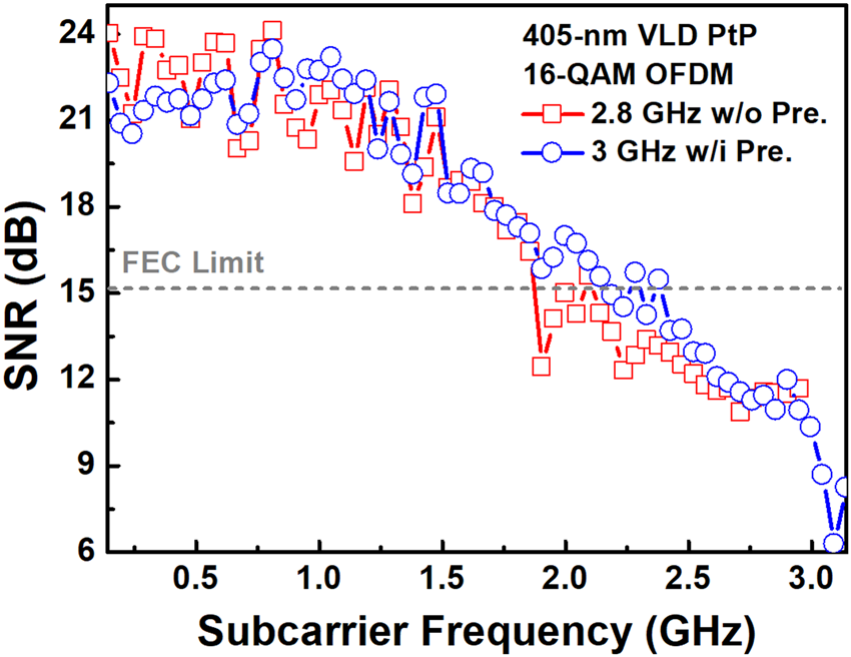
The VLD based PtP free-space data transmission. The constellation plots and related subcarrier SNRs of 11.2- and 12-Gbit/s 16-QAM OFDM data carried by the VLD after 7-m free-space transmission.

To perform high-quality data stream, the VLD needs not only large modulation bandwidth and small relative intensity noise (RIN), but also flat throughput intensity and high extinction ratio. The VLD optimizes its bias current to 75 mA for achieving a data rate of 11.2 Gbit/s under PtP transmission over a 7-m free-space link, which allows a 16-QAM OFDM data bandwidth of 2.8 GHz to meet the forward error correction (FEC) criterion, and the related error vector magnitude (EVM), signal-to-noise ratio (SNR) and bit-error-rate (BER) of 17.1%, 15.3 dB and 3.4 × 10^−3^ are observed, respectively, as shown in Fig. 1. To further upgrade the transmission capacity, increasing the OFDM data bandwidth induced high-frequency SNRs degradation can be compensated by introducing the pre-leveling technique^69^. In detail, the OFDM subcarrier pre-leveling technique is to pre-amplify the OFDM subcarriers by multiplying a rising exponential function^70^, which slightly sacrifices the subcarrier SNRs at low frequency to compensate the high-subcarrier-frequency SNRs for improving the average SNR of the transmitted QAM-OFDM data. This enlarges the allowable OFDM bandwidth to 3 GHz at a raw data rate of 12 Gbit/s, and the subcarrier SNRs and related constellation plot are also shown in Fig. 1 to reveal an average EVM of 17.2%, an average SNR of 15.2 dB and a BER of 3.8 × 10^−3^. Note that the transmission capacity as high as 12 Gbit/s is guaranteed for the VLD based 7-m PtP VLC system.

**Figure 2:**
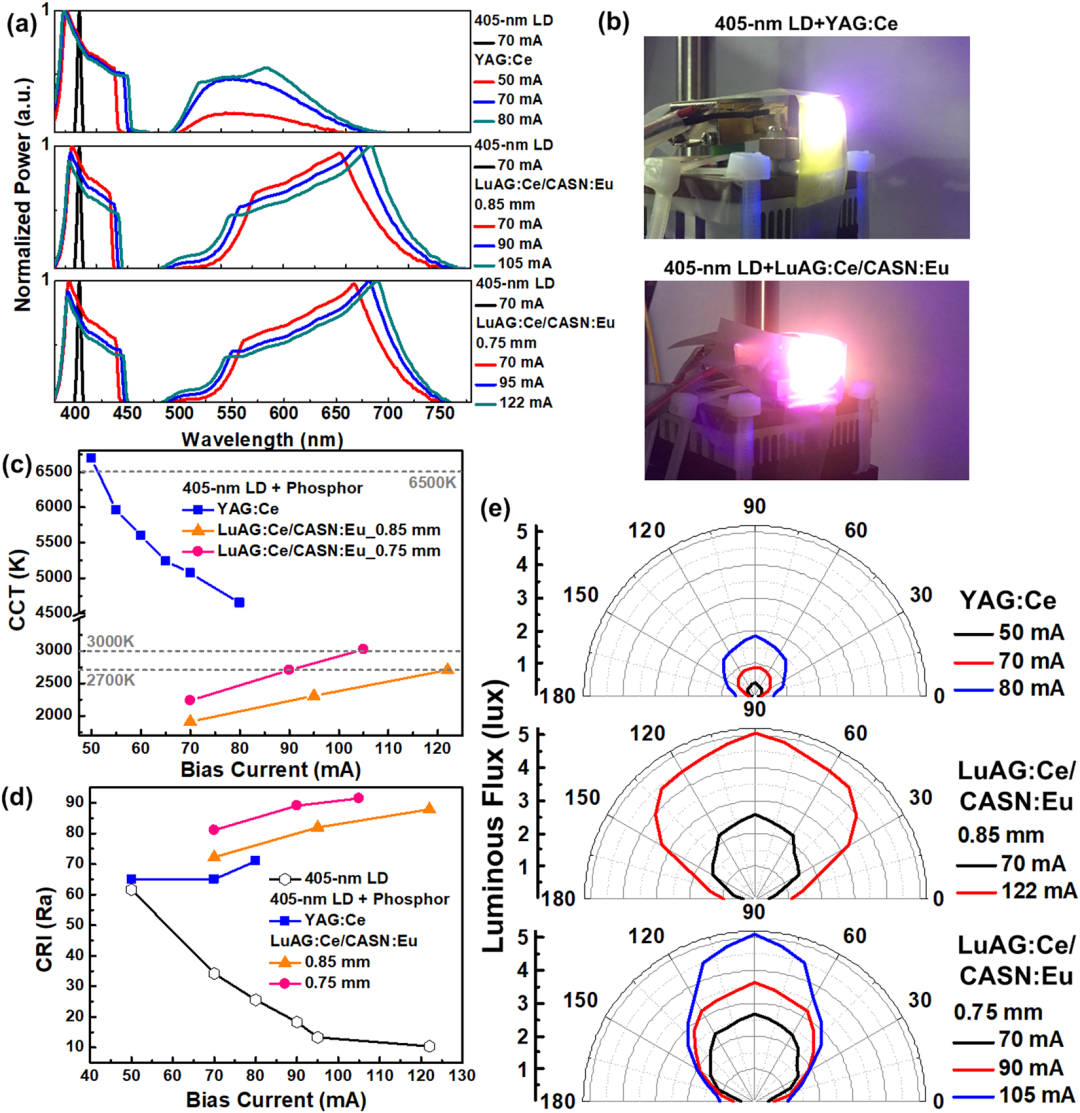
The white-lighting performances of the VLD illuminating YAG:Ce and LuAG:Ce/CASN:Eu phosphorous diffusers. (**a**) The optical spectra and (**b**) images of the white light generated by exciting the YAG:Ce and LuAG:Ce/CASN:Eu fluorescent phosphors with the violet laser beam. (**c**) The CCTs response of the VLD illuminating phosphor doped diffusers at different DC biases. (**d**) The CRIs performance of the VLD covered with either YAG:Ce or LuAG:Ce/CASN:Eu fluorescent phosphor. (**e**) The angle dependent white-light field distributions of the phosphorous diffusers divergent VLD.

For white-light generation, the VLD at different bias currents is employed to excite the YAG:Ce or LuAG:Ce/CASN:Eu fluorescent phosphor, and the corresponding optical spectra are shown in Fig. 2(a). By exciting the YAG:Ce or the LuAG:Ce/CASN:Eu fluorescent phosphor, the violet laser beam not only broadens its spectral linewidth but also emits blue-yellow or yellow-orange light, respectively. These excited light components are mixed with the divergent violet laser beam to generate the white light, as shown in Fig. 2(b). Note that the LuAG:Ce/CASN:Eu fluorescent phosphor enables to deliver the spontaneous emission light with longer wavelength and stronger intensity than the YAG:Ce does, indicating that the LuAG:Ce/CASN:Eu fluorescent phosphor provides higher exciting efficiency. Obviously, increasing the bias current of the VLD gradually broadens its spectral linewidth and enhances the excited light intensity for all cases.

Moreover, the Fig. 2(c) displays the achievable CCTs of the phosphorous diffuser covered VLD output at different bias currents, in which the link distance is 0.5 m. By increasing the bias current from 50 to 80 mA, the YAG:Ce fluorescent phosphor generated white light exhibits daylight appearance with its CCT reducing from 6750 to 4750 K. Such a variable CCT range entirely covers the International Commission on Illumination (CIE) defined standard illuminants of D50 (5000 K), D55 (5500 K) and D65 (6504 K). In contrast to the YAG:Ce fluorescent phosphor, the 0.85-mm-thick LuAG:Ce/CASN:Eu phosphorous diffuser covered VLD output enlarges its CCT from 1750 to 2700 K with increasing the bias current from 70 to 122 mA, and the proposed warm white-light source is comparable to commercial incandescent light bulbs. By thinning the phosphor thickness to 0.75 mm, it effectively reduces the demanded VLD bias current by 32 mA to deliver the white light with the same CCT of 2700 K. Enlarging the bias current to 105 mA slightly increases the CCT of the generated white light to 3000 K, which meets the CIE defined standard illuminant of F12. At the same bias current of 70 mA, the LuAG:Ce/CASN:Eu phosphorous diffuser covered VLD output clearly shows warmer white light than the YAG:Ce one, which verifies its higher exciting efficiency. In addition, the CRI responses of the white light generated from the VLD illuminated phosphor diffuser at different bias currents are shown and compared in Fig. 2(d). Without adding the phosphorous diffuser, the violet laser beam decreases its CRI from 62 to 10 by increasing the bias current from 50 to 122 mA. With the YAG:Ce fluorescent phosphor adhering to the front of the VLD, increasing the bias current from 50 to 80 mA effectively raises the CRI of the generated white light from 64 to 71. With the use of the 0.85-mm-thick LuAG:Ce/CASN:Eu fluorescent phosphor, an increased CRI from 72 to 88 is observed for the generated white light as the VLD bias current increases from 70 to 122 mA. At the same bias current of 70 mA, the 0.75-mm-thick LuAG:Ce/CASN:Eu phosphorous diffuser covered VLD output exhibits the highest CRI of 81 among all cases. Note that the CRI can further be increased up to 91.5 by continuously increasing the bias current of the VLD to 105 mA.

After 0.5-m propagation in free space, the angular radiation patterns of the phosphorous diffuser covered VLD output are shown in Fig. 2(e), where the 90° and 0° represent the normal and grazing incidences, respectively. Note that the highest luminance is obtained at an orientation angle of 90°, and the proposed white-light source significantly extends its optical field distribution by increasing the VLD bias current. Three cases show similar lambertian-like shape with uniform optical field distribution, which verifies the strong scattering effect of these phosphorous diffusers. By enlarging the bias current from 50 to 80 mA, the luminous flux of the VLD illuminating YAG:Ce phosphorous diffuser is increased from 0.4 to 1.8 lux with its optical field distribution ranging from 30° to 150°. At the same bias current of 70 mA, changing the phosphorous diffuser from YAG:Ce to the 0.85-mm-thick LuAG:Ce/CASN:Eu essentially enhances the luminous flux of the generated white light to 2.6 lux, which is slightly lower than that of 2.67 lux for the 0.75-mm-thick fluorescent phosphor delivered one. By increasing the bias current of the VLD to 122 mA, the luminous flux can be increased to 5.1 lux with its optical field distribution ranging from 25° to 155°. Thinning the thickness of the LuAG:Ce/CASN:Eu fluorescent phosphor to 0.75 mm and decreasing the bias current of the VLD to 105 mA make the generated white light remain a similar luminous flux of 5.2 lux. However, such a thickness induces relatively low scattering effect to exhibit a slightly focused optical field distribution ranged from 45° to 135°. As compared to the brightness of conventional white-light LEDs, although the luminous flux of proposed white light is somewhat low for lighting purpose, a LD array covered with phosphorous phosphor could help to solve this problem. Apparently, the residual violet light power is a simple multiplication with the number of LD chips in the array, as the generated white light has to preserve its CCT a constant during the multiplication of its illuminance. Assuming that the pupil diameter of the human eye is 7 mm and the free-space distance is 0.5 m, an irradiated angle of 0.8° (0.014 rad) with a related solid angle of 1.54 × 10^−4^ sr can be obtained for subsequent calculation^60^. After passing through the 0.85-mm-thick LuAG:Ce/CASN:Eu phosphorous diffuser, the divergent white light (without collimating and refocusing) of the VLD biased at 122 mA exhibits a residual power of only 0.43 μW (within an accepting aperture diameter of 1.2 mm) as mesured at a distance of 0.5 m from the source. This corresponds to a related irradiance of 3.8 mW/m^2^ for the residual violet light, indicating a radiance of 0.025 kW/m^2^sr to satisfy the IEC 62471 risk group-0 (RG0) criterion of < 0.1 kW/m^2^sr. That is, even a LD array with up to 400 chips can still pass the upper limitation of the RG1 criterion (0.1-10 kW/m^2^sr).

**Figure 3:**
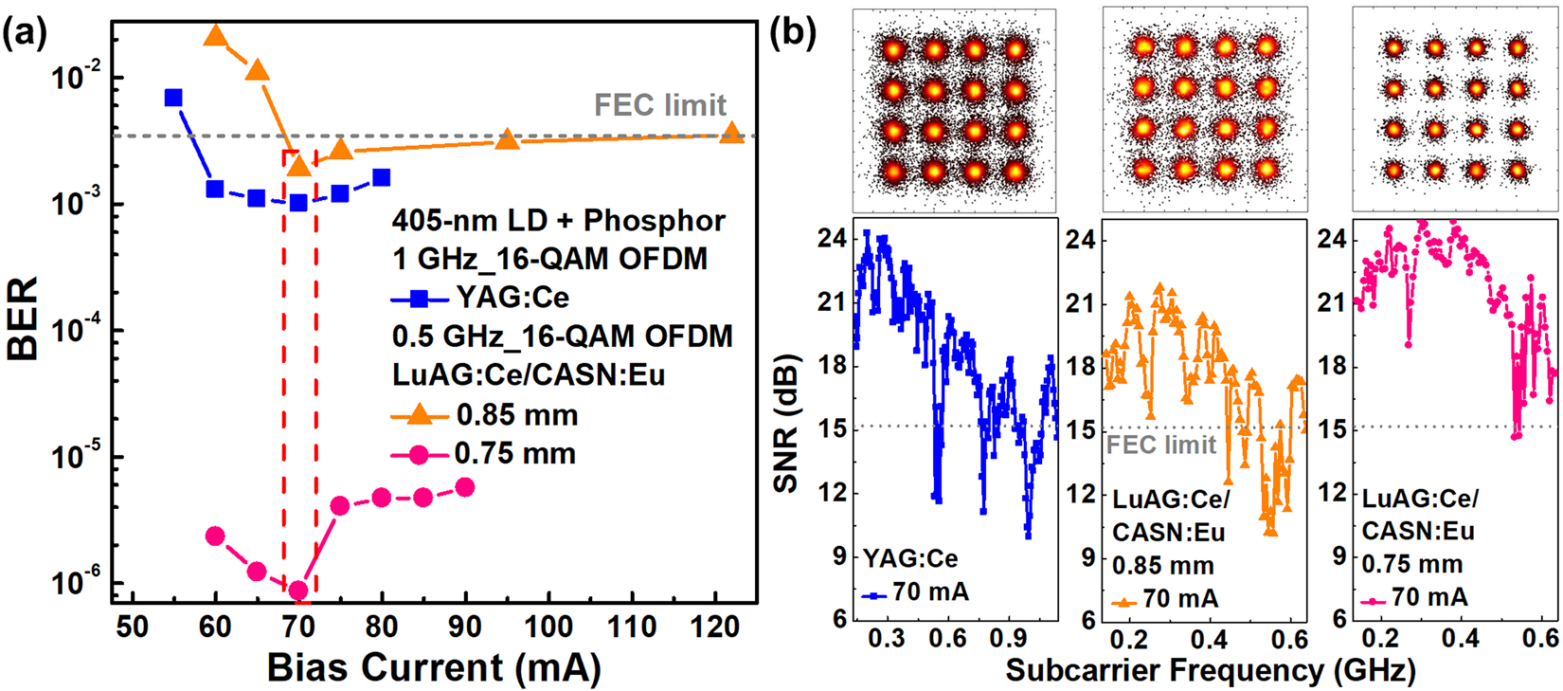
The operation optimization of the phosphorous diffusers divergent VLD. The BERs, constellation plots and subcarrier SNRs of 16-QAM OFDM data carried by the VLD illuminating phosphor doped diffusers at different bias currents.

To optimize the operational parameter for transmission after covering the VLD with the YAG:Ce and LuAG:Ce/CASN:Eu phosphorous diffusers, the VLD at different bias currents is directly encoded by two 16-QAM OFDM data with raw data rates of 4 and 2 Gbit/s covering bandwidths of 1 and 0.5 GHz, respectively. Indeed, the standard deviation of an OFDM signal can be determined to evaluate the clipping noise, which helps to optimize the operation condition of transmitters^71, 72^. In addition to the aforementioned standard deviation of the OFDM signal, the other parameters of the transmitter including its modulation bandwidth, throughput intensity, relative intensity noise, modulation depth and on/off extinction ratio should also be taken into account. As a result, the BERs, constellation plots and subcarrier SNRs of the 0.5-m free-space transmitted 16-QAM OFDM data carried by the phosphorous diffuser covered VLD at different bias currents are directly employed for optimization, as shown in Fig. 3. In principle, the insufficient bias current of the VLD induces inadequate modulation bandwidth, strong RIN and data clipping effect to distort the carried data and degrade its receiving performance^73, 74^. In contrast, the over biasing inevitably suffers from the declined throughput intensity at low frequency and the decreased data extinction, which also degrades the received data quality. When exciting the YAG:Ce and LuAG:Ce/CASN:Eu fluorescent phosphors with the divergent VLD beam, the same optimized bias current of 70 mA is observed to reveal the clearest constellation plot with the lowest BERs of 1 × 10^−3^, 8.63 × 10^−7^ (0.75-mm case) and 1.9 × 10^−3^ (0.85-mm case) for the carried 4- and 2-Gbit/s data, respectively. In detail, for the 4-Gbit/s data carried by the white light generated from the VLD excited YAG:Ce fluorescent phosphor, the average EVM of 14.9% and SNR of 16.5 dB are obtained. By using the 0.85-mm-thick LuAG:Ce/CASN:Eu fluorescent phosphor, the white light carried 2-Gbit/s data exhibits an average EVM of 15.9% and an SNR of 16 dB. In contrast, thinning the LuAG:Ce/CASN:Eu fluorescent phosphor to 0.75 mm effectively improves the EVM to 9.5% and the SNR to 20.5 dB.

**Figure 4:**
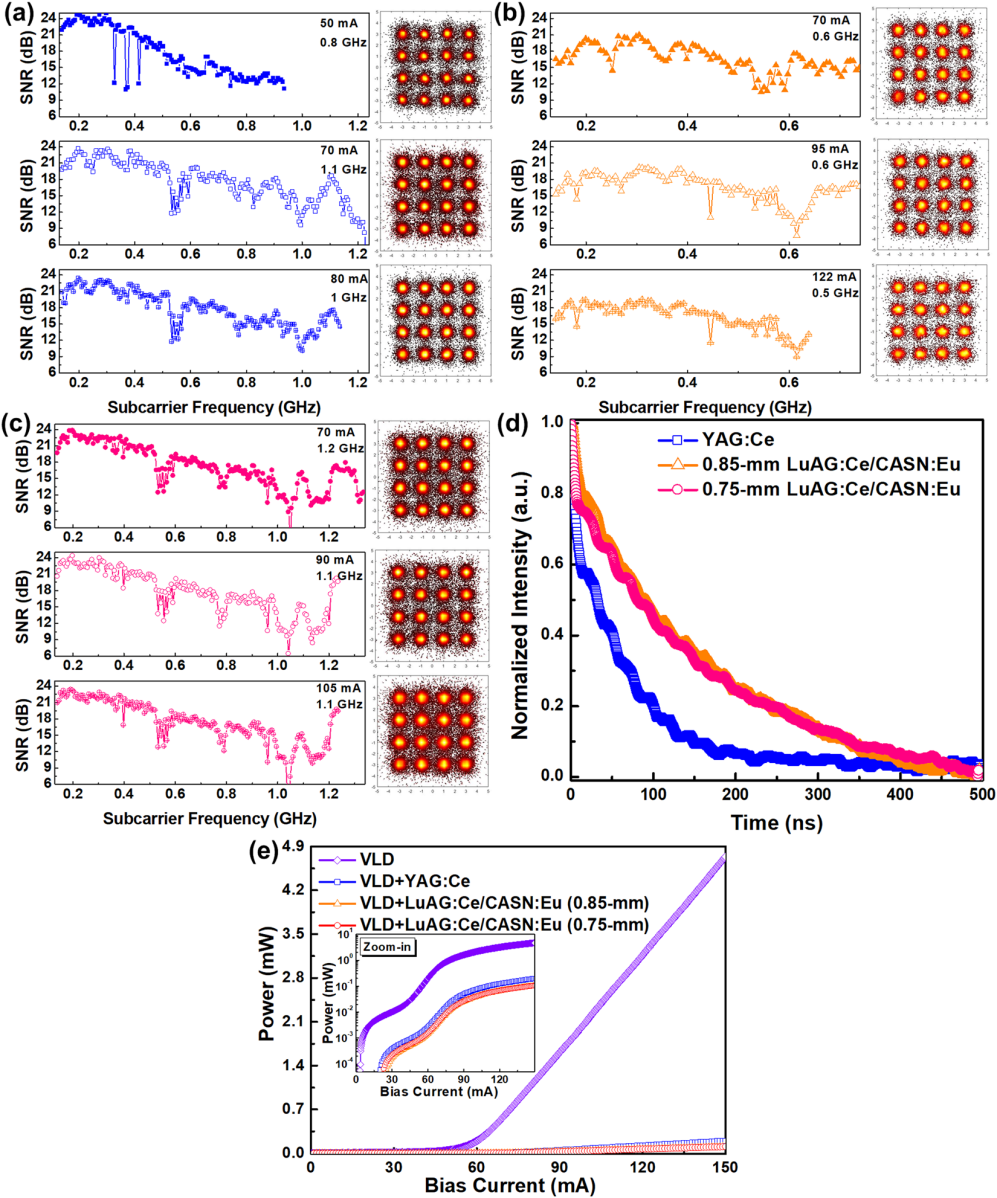
The transmission performances of proposed white-light source. The subcarrier SNRs and corresponding constellation plots of 0.5-m free-space transmitted 16-QAM-OFDM data carried by the VLD covered with (**a**) YAG:Ce, (**b**) 0.85-mm-thick LuAG:Ce/CASN:Eu and (**c**) 0.75-mm-thick LuAG:Ce/CASN:Eu fluorescent phosphors. (**d**) The TRPL spectrum of used YAG:Ce and LuAG:Ce/CASN:Eu fluorescent phosphors. (**e**) The violet power-to-current responses obtained without and with the YAG:Ce or LuAG:Ce/CASN:Eu phosphorous diffuser adhering to the front of the VLD.

For white-lighting communication, the VLD covered with YAG:Ce phosphorous diffuser changes its bias current to 50, 70 and 80 mA to obtain the corresponding allowable data rates, and the subcarrier SNRs and related constellation plots of received QAM-OFDM data are shown in Fig. 4(a). To meet the FEC demanded BER of 3.8 × 10^−3^ at a VLC bias current of 50 mA, the delivered white light successfully carries the 0.8-GHz 16-QAM OFDM data to achieve a raw data rate of 3.2 Gbit/s with an average EVM of 17.25%, an SNR of 15.3 dB and a BER of 3.6 × 10^−3^. Enlarging the bias current to 70 mA effectively extends the allowable OFDM data bandwidth to 1.1 GHz at a raw data rate of up to 4.4 Gbit/s, and the related EVM, SNR and BER of 16.4%, 15.7 dB and 2.5 × 10^−3^ are observed, respectively. By continuously increasing the bias current to 80 mA, although the declined throughput intensity of the VLD at low frequency is inevitably introduced, a raw data rate of 4 Gbit/s can still be achieved with an average EVM of 15.6%, an SNR of 16.1 dB and a BER of 1.6 × 10^−3^.

In contrast, by using the VLD to excite the 0.85-mm LuAG:Ce/CASN:Eu fluorescent phosphor for white-light generation, the allowable OFDM data bandwidth is slightly decreased from 1.1 to 0.6 GHz at the same bias current of 70 mA, which only achieves a raw data rate of 2.4 Gbit/s with corresponding EVM, SNR and BER of 16.8%, 15.8 dB and 3 × 10^−3^, respectively, as shown in Fig. 4(b). Enlarging the VLD bias current to 95 mA maintains the carried OFDM data bandwidth at same raw data rate of 2.4 Gbit/s with an EVM of 17%, an SNR of 15.4 dB and a BER of 3.3 × 10^−3^. By increasing the bias current up to 122 mA, the declined throughput of the VLD seriously decreases the allowable OFDM data bandwidth of the generated white light to 0.5 GHz, which limits the data rate to 2 Gbit/s with corresponding EVM, SNR and BER of 16.8%, 15.8 dB and 3.5 × 10^−3^, respectively.

For the 70-mA biased VLD covered with the 0.75-mm-thick LuAG:Ce/CASN:Eu fluorescent phosphor, a raw data rate as high as 4.8 Gbit/s with 16-QAM OFDM data covering 1.2-GHz bandwidth is observed with an EVM of 17.3%, an SNR of 15.2 dB and a BER of 3.7 × 10^−3^, as shown in Fig. 4(c). Increasing the bias current to 90 mA slightly decreases the allowable data rate to 4.4 Gbit/s, and the related EVM, SNR and BER of 16.9%, 15.5 dB and 3 × 10^−3^ are observed, respectively. When the bias current is further increased to 105 mA, the proposed white light maintains its transmission capacity at 4.4 Gbit/s with corresponding EVM, SNR and BER of 17.1%, 15.3 dB and 3.4 × 10^−3^, respectively. The last technique employed to compensate the high-frequency intensity declination and to extend the OFDM data bandwidth for upgrading the transmission capacity of the generated white light is the OFDM subcarrier pre-leveling technique. To pass the FEC criterion, the allowable OFDM data bandwidths (BW) and related raw data rates (D) of the VLD covered with phosphorous diffusers before and after pre-leveling are summarized in Table 1.

**Table 1:** The allowable 16-QAM OFDM data bandwidth and corresponding raw data rate of the VLD covered with YAG:Ce and LuAG:Ce/CASN:Eu phosphor doped diffusers before and after pre-leveling.

Phosphor	I_bias_ (mA)	BW (GHz)	D (Gbit/s)	Slope_Pre._ (dB/GHz)	BW (GHz)	D (Gbit/s)
YAG:Ce	50	0.8	3.2	0.8	0.9	3.6
	70	1.1	4.4	—	1.1	4.4
	80	1	4	0.8	1.1	4.4
LuAG:Ce/CASN:Eu 0.85 mm	70	0.6	2.4	0.2	0.7	2.8
	95	0.6	2.4	—	0.6	2.4
	122	0.5	2	0.6	0.6	2.4
LuAG:Ce/CASN:Eu 0.75 mm	70	1.2	4.8	—	1.2	4.8
	90	1.1	4.4	—	1.1	4.4
	105	1.1	4.4	—	1.1	4.4

Owing to the different operational conditions such as the bias current of the VLD and the various frequency responses induced by different phosphors, some of the cases cannot increase their raw data rates by using the pre-leveling technique. By pre-leveling with the same power-to-frequency slope of 0.8 dB/GHz, two cases of the YAG:Ce covered VLD biased at 50 and 80 mA effectively increase the allowable OFDM data bandwidths to 0.9 and 1.1 GHz at raw data rates of 3.6 and 4.4 Gbit/s, respectively. In contrast, at bias currents of 70 and 122 mA for the 0.85-mm-thick LuAG:Ce/CASN:Eu fluorescent phosphor covered VLD, the pre-leveling slopes of 0.2 and 0.6 dB/GHz help to increase the raw data rates of the generated white light to 2.8 and 2.4 Gbit/s, respectively.

In principle, the overall bandwidth may considerably be affected by both the spontaneous emission lifetime of the phosphor and the modulation bandwidth of the laser diode, depending on which mechanism dominates the systematic performance. To further clarify the effects of these phosphorous diffusers on the transmission capacity of the VLD based white-light source, the time-resolved photo-luminescence (TRPL) analyses for the YAG:Ce and LuAG:Ce/CASN:Eu fluorescent phosphors are performed and shown in Fig. 4(d). By fitting with an exponential function, a lifetime of 60 ns is obtained for the YAG:Ce fluorescent phosphor. In contrast, the 0.85- and 0.75-mm-thick LuAG:Ce/CASN:Eu fluorescent phosphors show their lifetimes of 130 and 120 ns, respectively. Note that these lifetimes are too long to support high-speed data transmission beyond Gbit/s, which means that the transmission capacity of the VLD based white-light source is irrelevant to the used phosphor but is strongly correlated with the residual violet laser beam. After collimating the beam for transmission and refocusing it for receiving, the residual VLD radiance within the proposed white light is calculated to estimate the induced hazard on the human eye. The power-to-current (P-I) responses of the residual violet light power are obtained without and with the YAG:Ce or LuAG:Ce/CASN:Eu phosphorous diffuser adhering to the VLD, as shown in 4(e). The VLD covered with phosphorous diffusers significantly decreases its P-I slope (*I > I*_*th*_) from 50 to around 2 mW/A as most of the violet laser light contributes to emit yellow-orange light. As compared to other cases, the 0.85-mm-thick LuAG:Ce/CASN:Eu phosphorous diffuser covered VLD at a bias current of 122 mA exhibits the maximal power of 0.07 mW with a related irradiance of 0.62 W/m^2^ for the residual violet light. This indicates a radiance of 4.026 kW*/*m^2^sr to satisfy the RG1 criterion.

For comparison, the white-lighting and data transmission performances of the VLD covered with YAG:Ce and LuAG:Ce/CASN:Eu phosphorous diffusers at different operation conditions are summarized in Table 2. As a result, the use of YAG:Ce fluorescent phosphor enables to generate the white light with its color temperature fitting the daylight appearance, and the corresponding CCT can be decreased to provide warm white light by increasing the bias current of the VLD. When biasing the VLD at 70 mA, the generated white light with a CCT of 5068 K and a CRI of 65 achieves 4.4-Gbit/s raw data rate. In comparison with the YAG:Ce fluorescent phosphor, adhering the LuAG:Ce/CASN:Eu fluorescent phosphor to the VLD ensures the delivered white light a warm-white color with lower CCT, comparable transmission capacity and higher CRI. To achieve a CRI of 2700 K, the VLD illuminating 0.85-mm-thick LuAG:Ce/CASN:Eu phosphor doped diffuser is required to increase its bias current to 122 mA, and the generated white light with a CRI of 87.9 can support a raw data rate of 2.4 Gbit/s. The thinner LuAG:Ce/CASN:Eu fluorescent phosphor enables the white light to exhibit higher CCT, CRI and transmission capacity. At the same bias current of 70 mA, the VLD illuminating 0.75-mm-thick LuAG:Ce/CASN:Eu fluorescent phosphor provides the largest transmission capacity of 4.8 Gbit/s, the highest CRI of 81.1 and the lowest CCT of 2235 K when comparing with other cases. For the LuAG:Ce/CASN:Eu phosphorous diffusers with different thicknesses, the thinner one has higher SNR to achieve higher data rate than the thicker one. To increase the CCT to 3000 K for competing with the commercial incandescent light bulb, the VLD needs to enlarge its bias current to 105 mA for the generated white light with its CRI enlarging up to 91.5 at a cost of slightly decreasing the transmission capacity to 4.4 Gbit/s. The use of VLD combined with the different fluorescent phosphors and the pre-leveling technique effectively implements the low-CCT and high-CRI white-lighting communication, which is a powerful candidate for next-generation indoor lighting industries.

**Table 2:** The white-lighting and data transmission performances of the VLD covered with YAG:Ce and LuAG:Ce/CASN:Eu phosphorous diffusers.

Phosphor	I_bias_ (mA)	CCT (K)	CRI	BW (GHz)	SNR (dB)	D (Gbit/s)
YAG:Ce	50	6696	64.9	0.9	15.6	3.6
	70	5068	65	1.1	15.7	4.4
	80	4650	70.9	1.1	15.5	4.4
LuAG:Ce/CASN:Eu 0.85 mm	70	1912	72.7	0.7	15.2	2.8
	95	2307	82	0.6	15.4	2.4
	122	2706	87.9	0.6	15.6	2.4
LuAG:Ce/CASN:Eu 0.75 mm	70	2235	81.1	1.2	15.2	4.8
	90	2703	89.1	1.1	15.5	4.4
	105	3023	91.5	1.1	15.3	4.4

## Conclusion

Violet laser enabled warm white light or daylight is performed to realize the high-speed VLC. With adhering the LuAG:Ce/CASN:Eu or YAG:Ce phosphorous diffuser to the VLD, the high-CRI white-lighting and high-spectral-usage data transmission can meet the demand of smart lighting communication for green housing applications. The transmission capacity as high as 12 Gbit/s over 7 m for the VLD based PtP VLC system is obtained. When covering the YAG:Ce phosphorous diffuser to the front of the VLD biased at 70 mA, the white light with a CCT of 5068 K and a CRI of 65 enables to support 4.4-Gbit/s data rate with FEC certified SNR, EVM and BER of 15.7 dB, 16.4% and 2.5 × 10^−3^, respectively, after 0.5-m free-space transmission. When replacing the YAG:Ce plate with the 0.85-mm-thick LuAG:Ce/CASN:Eu plate for the VLD white-lighting at the same bias current, the delivered white light increases its CRI to 72.7 at a cost of decreasing CCT and transmission capacity to 1912K and 2.8 Gbit/s, respectively. To emphasize the CCT of the generated white light at 2700 K for competing with the commercial incandescent light bulb, the demanded bias current of the VLD must increase up to 122 mA, thus providing an increased CRI of 87.9 and a decreased data rate of 2.4 Gbit/s. Thinning the thickness of the LuAG:Ce/CASN:Eu phosphorous diffuser to 0.75 mm is an efficient way to maintain the same CCT value but enlarges the transmission capacity for the proposed white light. By decreasing the required VLD bias current by 32 mA, the proposed white-light source increases its CRI up to 89.1 and supports the data rate as high as 4.4-Gbit/s, which provides the FEC qualified SNR, EVM and BER of 15.5 dB, 16.9% and 3 × 10^−3^, respectively. By further enlarging the bias current of the VLD to 105 mA, the proposed white-light source maintains its transmission capacity at 4.4 Gbit/s to provide a slightly increased CCT of 3023 K and a CRI of up to 91.5. This work declares two different phosphorous diffusers to enable the VLD white-lighting with diverged spot, low CCT and high CRI for high-speed data transmission, which could play important role in next-generation indoor lighting industry.

## Methods

**Figure 5:**
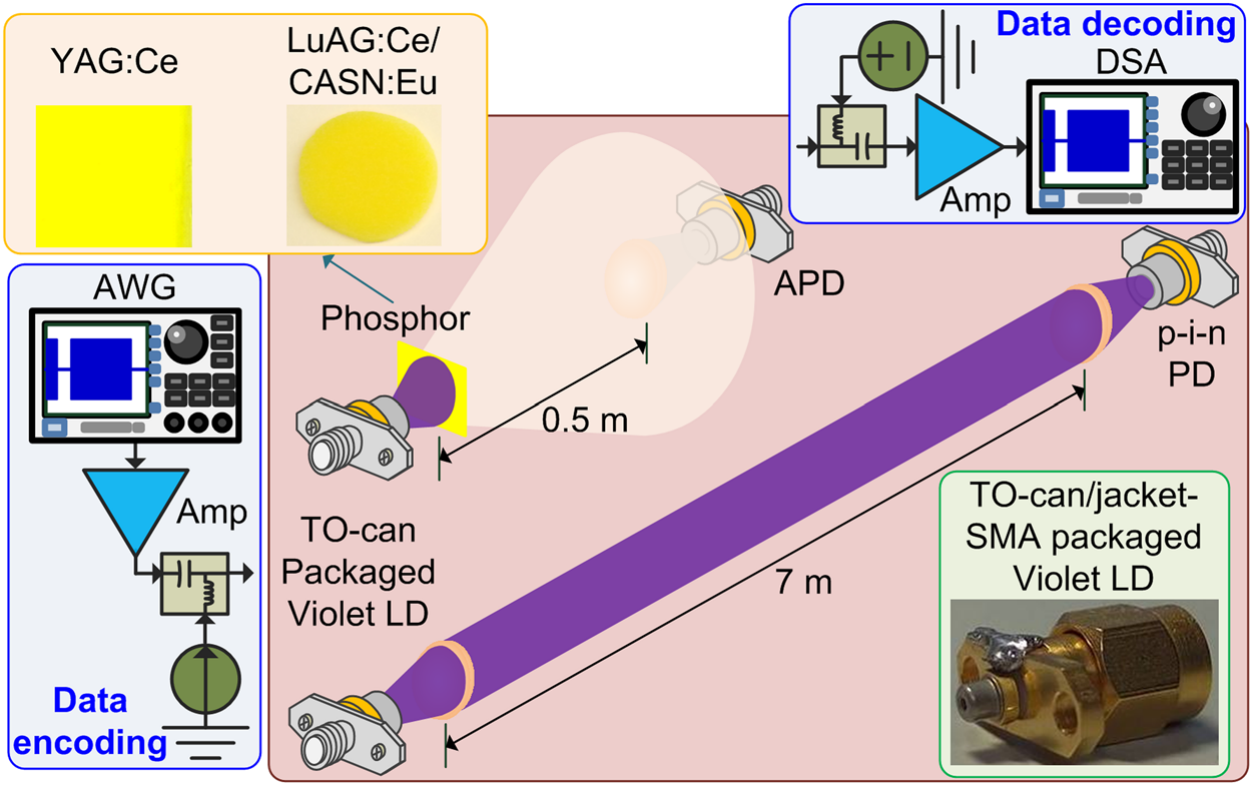
The schematic diagram of VLD based PtP and white-lighting communication systems. The VLD based PtP and white-lighting communications over the 7-m and 0.5-m free-space links, respectively.

The infrastructures for implementing the VLD based PtP and white-lighting communications were illustrated in Fig. 5, in which the used VLD with a TO-can package exhibits a peak wavelength of 404 nm, a threshold current (*I*_*th*_) of 34 mA and a power-to-current slope of 1.23 W/A (*I*_*bias*_ > *I*_*th*_). A pair of plano-convex lenses with the same focal length of 10 mm and numerical aperture of 0.3 helps to control the laser beam divergence, the collimation during free-space transmission and the convergence of beam prior to the receiver. A Si p-i-n photodiode (PD, Thorlabs, FDS025) with a 3-dB bandwidth of 3.3 GHz was employed for PtP data receiving. For white-light generation, either the YAG:Ce^56^ or the LuAG:Ce/CASN:Eu^75^ luminescent phosphor was individually adhered to the VLD, and an avalanche photodiode (APD, Hamamatsu, S12023-02) with a cut-off frequency of 1 GHz was used for receiving the optical data. To suppress the parasitic capacitance and inductance induced declination on frequency response, the pin lengths of the used VLD and PDs are shortened to 3 mm before connecting with the SubMiniature version A (SMA) jacks. A homemade temperature controlling system with electrical isolation was assembled to stabilize the output dynamics of the VLD and PDs^54^.

For directly encoding the VLD, the electrical QAM-OFDM data with various bandwidths are delivered by importing a homemade MATLAB programing data into an arbitrary waveform generator (AWG, Tektronix, 70001 A) with a sampling rate of 24 GSa/s. Afterwards, the output waveform after passing through an electrical amplifier (Amp, Picosecond 5828 A with a gain of 10 dB for the PtP case and Picosecond 5865 with a gain of 26 dB for the white-lighting case) was carried by the VLD for data transmission. The optical transmission distances for the PtP and white-lighting communications were set as 7 and 0.5 m, respectively. After receiving with the p-i-n PD for PtP case and the APD for white-lighting case, the optoelectrically converted QAM-OFDM data was amplified by another amplifier with 18-dB gain (New focus, 1422) for PtP case and 40-dB gain (Mini-circuit, ZKL-1R5+) for white-lighting case, respectively. Then the amplified waveform was captured by a 100-GSa/s digital serial analyzer (DSA, Tektronix, 71604 C), and a decoded MATLAB program was employed to evaluate the QAM-OFDM data qualities including constellation plot, EVM, SNR and BER.
